# Factors associated with risk related to the use of psychoactive substances by men deprived of their liberty

**DOI:** 10.1590/1518-8345.5972.3669

**Published:** 2022-10-07

**Authors:** Wanessa Cristina Baccon, Maria Aparecida Salci, Aroldo Gavioli, Magda Lúcia Félix de Oliveira, Francielle Renata Danielli Martins Marques, Priscila Garcia Marques

**Affiliations:** 1 Universidade Estadual de Maringá, Maringá, PR, Brazil.; 2 Bolsista da Coordenação de Aperfeiçoamento de Pessoal de Nível Superior (CAPES), Brazil.

**Keywords:** Mass Screening, Substance-Related Disorders, Prisoners, Prisons, Illicit Drugs, Public Health Nursing, Programas de Rastreamento, Transtornos Relacionados ao Uso de Drogas, Prisioneiros, Prisões, Drogas Ilícitas, Enfermagem em Saúde Pública, Tamizaje Masivo, Trastornos Relacionados con Sustancias, Prisioneros, Prisiones, Drogas Ilícitas, Enfermería en Salud Pública

## Abstract

**Objective:**

to evaluate the factors associated with risk related to the use of psychoactive substances in male inmates of a prison in a city in the South of Brazil.

**Method:**

a cross-sectional data from 220 men deprived of liberty, inmates of a provisional custody institution in the State of Paraná, collected with a screening instrument and questionnaire. Binary logistic regression and odds ratio analysis were used to verify associations between risk related to substance use and socio-demographic characteristics of living conditions before incarceration and current incarceration.

**Results:**

the adjusted model revealed association of consumption with skin color brown/black and yellow, those who had only one parent responsible until age 15, age at first arrest 18 or older, professing religion, working before arrest, owning their own house, living alone, receiving visitors in prison.

**Conclusion:**

the identified factors are useful to insert effective treatment proposals and reduce the gaps and social vulnerability existing in prison.

## Resumo

**Objetivo:**

avaliar os fatores associados ao risco relacionado ao uso de substâncias psicoativas em homens internos de uma prisão em um município do Sul do Brasil.

**Método:**

dados transversais de 220 homens privados de liberdade, internos de uma instituição de custódia provisória no Estado do Paraná, coletados com instrumento de rastreamento e questionário. Utilizaram-se análise de regressão logística binária e razão de chances para verificar associações entre o risco relacionado ao uso de substâncias psicoativas e as características sociodemográficas das condições de vida antes do encarceramento e do encarceramento atual.

**Resultados:**

o modelo ajustado revelou associação do consumo com a cor da pele parda/preta e amarela, aqueles que tiveram apenas um dos pais responsável até os 15 anos de idade, idade da primeira prisão com 18 anos ou mais, professar religião, trabalhar antes da prisão, moradia própria, morar sozinho, receber visitas na prisão.

**Conclusão:**

os fatores identificados são úteis para inserir propostas de tratamento eficazes e reduzir as lacunas e a vulnerabilidade social existentes na prisão.

## Resumen

**Objetivo:**

evaluar los factores asociados al riesgo relacionado con el uso de sustancias psicoactivas en hombres internados en una prisión, en un municipio en el Sur de Brasil.

**Método:**

datos transversales de 220 hombres privados de libertad, internados en una institución de custodia provisional, en el estado de Paraná, recolectados con instrumento de rastreo y cuestionario. Se utilizó el análisis de regresión logística binaria y la razón de chances para verificar asociaciones entre el riesgo relacionado con el uso de sustancias psicoactivas y las características sociodemográficas, de las condiciones de vida antes del encarcelamiento y del encarcelamiento actual.

**Resultados:**

el modelo ajustado reveló asociación del consumo con: el color de la piel marrón/negra y amarilla; aquellos que tuvieron apenas uno de los padres responsables hasta los 15 años de edad; edad de la primera prisión con 18 años o más; profesar religión; trabajar antes de la prisión; residencia propia; vivir solo; y, recibir visitas en la prisión.

**Conclusión:**

los factores identificados son útiles para introducir propuestas de tratamientos eficaces y reducir los vacíos y la vulnerabilidad social existentes en la prisión.

**Highlights**

(1) Significant results between characteristics of PDLs and risks related to their use.

(2) Marijuana was the illicit drug most consumed by person deprived of liberty.

(3) Associations between cocaine/crack with living alone and age of first arrest.

(4) Self-reported skin color brown/black and yellow predominated in this study.

(5) Associations between age and family structure up to age 15 with hypnotic use.

## Introduction

The prison environment has occupied a prominent place in public policies worldwide due to the rapid pace of growth of the prison population. In the world, there are more than ten million people living in prison, and Brazil ranks third among the countries with the largest prison populations in the world, with about 730,000 persons deprived of liberty (PDLs)^1^ ^-^ ^2^.

Investigating the health conditions of PDLs represents a challenge to researchers, since this population is considered by public opinion as undeserving of any assistance, and the problems identified are seen as punishment for previous acts. However, it is understood that a hostile and unhealthy prison environment can hinder the subsequent re-socialization process^3^.

The prison environment is considered detrimental to the physical and emotional health conditions of PDLs, culminating not only in the deprivation of liberty, but also of dignity^4^. Prisons have overcrowded facilities, increasing the risk of exposure to various untreated or undetected pathologies, violence, and psychoactive substance abuse (PASs)^5^.

Lifelong use of illicit substances and the consequent chemical dependency are a reality for more than 50% of PDLs^6^ ^-^ ^7^. Many PDLs report being under the influence of PAS at the time they committed the crime for which they were arrested or that the reason for arrest was related to offenses related to trafficking, possession or consumption of PASs^8^.

As a consequence, the worldwide rate of chemical dependency-related mental disorders from PASs has increased significantly in recent decades, reaching approximately 29.5 million people in 2015^9^. In this context, it is observed that PASs are often present in prison systems around the world, being particularly used at the beginning, as a way for PDLs to deal with the evils of incarceration, such as overcrowding, unhealthy conditions, exposure to violence, lack of health care and the breakdown of family ties^10^ ^-^ ^12^. With continued and prolonged use, PASs cease to be a means to survival and the consequent development of addiction becomes an end in itself^13^.

It is a consensus in the national and international literature that the addicts of PAS are overrepresented in the prison populations and with similar characteristics: low socioeconomic status, low education and with physical and mental health problems^12^ ^,^ ^14^ ^-^ ^16^. Because incarceration represents a constant challenge to be faced by PDLs, the public health field must be concerned with prisons as a cause of health inequities^16^.

Economic, family, housing, skin color, age, among others, are social determinants of health that directly impact the PDLs^17^ ^-^ ^18^. Therefore, the relationships between incarceration, PASs, and social determinants of health are urgently needed evidence to improve quality of life and subsequent re-socialization for PDLs^17^. Although substance abuse in PDLs is estimated to be ten times more prevalent than in the general population, problems with these substances are not always detected in prisons^12^. In addition, the perception of belonging to social groups excluded from most of the benefits of a population generates feelings of inferiority, suffering, and discrimination, which directly influence individual choices about health.

Given this problem and the high presence of chemical dependency in PDLs, in 2014, the National Policy of Integral Attention to the Health of Persons Deprived of Liberty in the Prison System (PDLPS) was instituted, which reformulated the composition of health teams in the prison system and expanded the scope of action to the specifics of mental health^19^. 

It is known that the consumption of PASs is allowed in the prison environment in the case of prescribed psychotropic drugs and tobacco, but any other PASs are prohibited during incarceration^4^. However, data on this consumption in PDLs is still quite scarce. It is believed that the lack of information on its circulation and consumption in prisons may be related to the complexity of discussing these phenomena in public security institutions^10^. Because it is a veiled and denied situation, it is believed that PDL do not receive mental health care suited to the premises of harm reduction because the data on the number of PAS users in prisons and the types of PASs used are most likely under-reported^13^.

Given this context, it is critical to identify predictors and risk factors for PAS use by PDLs in order to correct the current paucity of literature and inform appropriate prevention and harm minimization responses. Knowledge of predictors and risk factors for PAS use by PDLs may allow for the prediction of PAS use consequent to the generation of data used to inform specific policy and prevention options for high consumption in PDLs. 

Therefore, the objective of this study was to evaluate the consumption of PAS and associated factors among male prisoners in a prison in a city in the South of Brazil.

## Method

### Study design

This is a cross-sectional study, carried out with men deprived of liberty, inmates of a provisional maximum security prison unit in a city in northwestern Paraná. The guidelines for strengthening the reporting of observational studies in epidemiology (STROBE) were followed^20^.

### Setting where the data collection took place

The research setting was a temporary custody house in a medium-sized municipality located in the northwest of the State of Paraná, Brazil. The institution was opened in 2008, being a maximum security penal establishment whose purpose is to allocate vacancies only to provisional PDLs awaiting criminal conviction, specifically for the male population. However, due to the shortage of vacancies in the state penitentiary of reference, due to overcrowding, the penal unit absorbs provisional PDLs and also those already convicted. 

### Period

Data was collected in the months of June to November 2019, in the morning and afternoon periods.

### Population

The study selected men deprived of their freedom who were inmates of a provisional custody institution. In the month before data collection started, the unit housed 1183 inmates; 535 were convicted and 648 were temporary. 

### Selection criteria 

As the house of custody is intended for PDLs without criminal conviction, we considered as a selection criterion only men in provisional regime (prison management software SPR, v2). 

Those with clinical diagnoses related to Neurology and Psychiatry and/or with cognitive limitations that hindered communication and responses to the interviews were excluded (4) and with a prison time of less than 25 days (1).

### Sample definition

With the list made available by the institution containing all men deprived of liberty and considering that the population is finite (643 PDLs), stratified sampling was carried out, with an estimation error of 5%, Confidence Interval of 95% and prevalence of 30%^21^ ^-^ ^23^, resulting in a minimum sample size of 216 people.

After the sample calculation, a random and stratified drawing was made of 160 cells that house, on average, eight people. This way, all the PDLs considered as provisional had chances to belong to the sample and, at the end of the selection process, the final sample of the study consisted of 220 people.

### Study variables

To assess the risk related to the use (RRU) of PASs in PLWH, information was collected regarding three groups of independent variables. The first group contained the sociodemographic characteristics: age in years and categorized (19 to 39 years and 40 to 64 years); self-reported skin color, which was classified as white and other (brown/black or yellow); having a partner (no and yes); religious beliefs (yes and no), and children (yes and no). In the second group, living conditions before imprisonment were considered: housing condition, classified as owned, rented, and others (borrowed, relatives, or homeless); lived alone (yes and no); worked before imprisonment (yes and no); family income (no income and with income); family member responsible until age 15, categorized as both parents, only one parent, and others (other relatives or none), and age at first arrest (12 to 17 years and 18 years or older). The last group presented the characteristics of the current incarceration: reason for the current arrest, categorized in PASs trafficking and/or association to trafficking and others (assault, robbery, theft, receiving stolen goods, homicide, sex crime, domestic violence, and counterfeiting currency); length of current arrest (up to one year and more than one year) and whether they receive visitors (yes and no).

### Data collection instrument

A structured interview script, on paper and pen, was administered to all participants, taking approximately 40 minutes to complete. It was composed of two modules: the first module presented the characteristics of the PDLs divided into three groups (socio-demographic, living conditions before incarceration and current incarceration); the second module was the screening instrument Alcohol, Smoking and Substance Involvement Screening Test (ASSIST version 3.1), developed by the World Health Organization (WHO), translated and validated in Brazil, used for the screening and diagnosis of the level of RRU and the dependence on tobacco products, alcoholic beverages, marijuana, cocaine/crack, amphetamines or ecstasy, inhalants, hypnotics/sedatives, hallucinogens, opioids/opiates, injectables, and other PASs^24^ ^-^ ^25^.

The instrument was adapted to the Brazilian culture and is composed of eight questions, easy to apply, which address the frequency of PAS use in life and in the last three months, problems related to use, concern about the use by people close to the user, impairment in the execution of expected tasks, unsuccessful attempts to stop or reduce use, feeling of compulsion, and injecting use. Each response corresponds to a score, the sum total of which can range from zero to 39. The score results in the RRU of the screened PASs. Low risk (score of zero to ten for alcohol and zero to three for other PAS) is considered occasional use, indicating no intervention. Moderate risk (score of four to 26) is indicative of abuse, and brief intervention and/or counseling is recommended. High risk (score of 27 or higher) is suggestive of dependence, with referral to intensive treatment^24^ ^-^ ^26^. After applying the instrument, individuals who have never used any of the PASs are considered to be at no risk.

### Data collection

Data collection was carried out by a single researcher, a nurse from the Postgraduate Program in Nursing of the State University of Maringá, with specific training and authorization to carry out data collection inside the prison institution. The prison staff did not participate in the recruitment or data collection process, and had no knowledge of participation or response rates. The interviews were conducted in the health sector of the facility, in a private room, where only the researcher and the detainee were present. For security reasons, the door remained open and the prison guard stood outside the room. The research was done by means of an individual interview, conducted with two instruments, as described above.

### Data treatment and analysis

After collecting the information, the data were compiled in electronic spreadsheets. Next, descriptive analysis (mean, standard deviation, median, and absolute and relative frequencies) was performed for the variables that characterized the PDLs (sociodemographic, living conditions before incarceration and current incarceration).

The presence (low, moderate, and high) or absence (none) of the RRU of PASs in PDLs after ASSIST screening was considered as the outcome variable. Univariate and multiple binary logistic regression models were employed to determine the factors associated with the presence of RRU. The stepwise both method was used for the selection of variables and fitting of the final models. The adequacy of these models was verified with the analysis of quantile randomized residuals^27^ and collinearity was tested with the variance inflation factor (VIF). Associations were estimated by calculating the odds ratio (OR), adopting the 95% Confidence Interval (CI) as a measure of accuracy^28^. The analyses were performed in R software, version 4.0.4^29^.

### Ethical aspects 

After the appreciation of the Permanent Committee for Ethics in Research with Human Beings (COPEP) of the State University of Maringá (PR) (Opinion no. 3.211.746/2019), the research was approved with Certificate of Ethical Appreciation Submission (CAAE) number 08936619.4.0000.0104, on March 20, 2019, and complied with all the ethical precepts of Resolutions no. 466/2012 and no. 510/2016 of the National Health Council. It is noteworthy that there were no refusals and all participants signed the Free and Informed Consent Term (FICT).

## Results

The mean age (years) of males was 30.9, with a standard deviation of 10.1 and a median of 29, with a minimum age of 19 and a maximum of 64. There was a predominance of adult subjects, aged 19 to 59 years (n=216), when compared to the elderly, aged 60 to 64 years (n=4). The sociodemographic profile and living conditions before incarceration and current incarceration of the 220 men are detailed in Table 1.

The color self-reported by 61.8% of the PDLs was other, being 133 black/black and three yellow, 50.5% had no partner, and the majority (94.5%) professed religious belief and had children (65.9%). With regard to living conditions before incarceration, 60% lived in their own homes, 82.7% did not live alone, 89.1% worked before incarceration, 93.2% had a paid job before incarceration, 45% had only one parent as the responsible until age 15, and 76.8% were 18 years old or older at the age of first imprisonment.

When compared to the other reasons for imprisonment, drug trafficking and/or association to drug trafficking was the most common type (39.5%) of crime that led to imprisonment in this sample, and, because it is a provisional custody institution, most prisoners (71.4%) had up to one year of imprisonment and 60.9% received visits.

**Table 1:** Sociodemographic characterization, living condition before incarceration and current incarceration of person deprived of liberty (n=220). Maringá, PR, Brazil, 2019

Characteristics of PDLs^*^		n (%)
Variables	Categories	
**Sociodemographic**		
Age	19 to 39 years old	115 (52.3)
	40 to 64 years old	105 (47.7)
Skin color	White	84 (38.2)
	Other	136 (61.8)
Has a partner	No	111 (50.5)
	Yes	109 (49.5)
Professes religion	No	12 (5.5)
	Yes	208 (94.5)
Children	No	75 (34.1)
	Yes	145 (65.9)
**Living conditions before incarceration**		
Housing conditions	Owned	132 (60.0)
	Rented	74 (33.6)
	Other	14 (6.4)
Lived alone	No	182 (82.7)
	Yes	38 (17.3)
Worked	No	24 (10.9)
	Yes	196 (89.1)
Family income	Without income	15 (6.8)
	With income	205 (93.2)
Family member responsible until age 15	Both parents	82 (37.3)
	Only one of the parents	99 (45.0)
	Others	39 (17.7)
Age of first detention	12 to 17 years	51 (23.2)
	18 years or more	169 (76.8)
**Current incarceration**		
Reason for Detention	Dealing^†^	87 (39.5)
	Others	133 (60.5)
Prison time	Up to one year	157 (71.4)
	More than one year	63 (28.6)
Visitation	No	86 (39.1)
	Yes	134 (60.9)

* Persons deprived of liberty; ^†^Trafficking in SPAs and/or association to trafficking

Table 2 presents the characterization of use, i.e., those drugs that have been at least once tried by the PDLs and the RRU classification of PASs screened by ASSIST. The data on the current use of PASs consumed by PDLs and the RRU classification showed that 79.5% of men were screened as users of tobacco products, distributed as follows: 9.5% were classified as low risk, 59.5% as moderate risk, and 10.5% as high risk. For the RRU for alcoholic beverages, use was observed in 97.7% of men, of which 85.9% were classified as low risk, 9.5% as moderate risk, and 2.3% as high risk.

For illicit PAS, the following pattern of distribution of the RRU classification was observed: for marijuana, 72.3% with sustained use, 12.3% being low risk, 55.0% moderate risk, and 5.0% high risk. For cocaine/crack, 60% had current sustained use, 9.1% being low risk, 48.6% moderate risk, and 2.3% high risk. Amphetamines or ecstasy were used by 33.2%, with 19.1% being low risk, 14.1% moderate risk, and no high RRU for this substance. We observed that six men (2.7%) reported having used PASs by injection on an experimental basis.

**Table 2:** Characterization of lifetime use and risk rating related to use (RRU) of psychoactive substances (PASs), screened by ASSIST 3.1 in person deprived of liberty (PDLs) (n=220). Maringá, PR, Brazil, 2019

Psychoactive Substances (PASs)	Use in life*	Risk Related to Use (“RRU”) Level			
		Non-user	Low	Moderate	Elevated
	n (%)	n (%)	n (%)	n (%)	n (%)
Tobacco products	175 (79.5)	45 (20.5)	21 (9.5)	131 (59.5)	23 (10.5)
Alcoholic beverages	215 (97.7)	5 (2.3)	189 (85.9)	21 (9.5)	5 (2.3)
Marijuana	159 (72.3)	61 (27.7)	27 (12.3)	121 (55.0)	11 (5.0)
Cocaine/crack	132 (60.0)	88 (40.0)	20 (9.1)	107 (48.6)	5 (2.3)
Amphetamines or ecstasy	73 (33.2)	147 (66.8)	42 (19.1)	31 (14.1)	0 (0.0)
Inhalants	93 (42.3)	127 (57.7)	64 (29.1)	29 (13.2)	0 (0.0)
Hypnotics/sedatives	71 (32.3)	149 (67.7)	24 (10.9)	42 (19.1)	5 (2.3)
Hallucinogens	80 (36.4)	140 (63.6)	53 (24.1)	27 (12.3)	0 (0.0)
Opioids/opiates	9 (4.1)	211 (95.9)	7 (3.2)	2 (0.9)	0 (0.0)

*Psychoactive substances that have been tried at least once by PDLs

Table 3 presents the presence of the RRU of PASs according to sociodemographic characteristics and living conditions before incarceration and current incarceration according to each PAS screened by ASSIST 3.1. The percentage of RRU of all screened PASs for brown/black and yellow skin color stands out, except for opioids/opioids. Most had a partner, professed religion, had children, owned their own house, did not live alone, worked, and had an income. Regarding the family member responsible until age 15, the prevalence of RRU for the screened PASs was for only one parent responsible until age 15, except for opioids/opioids.

**Table 3:** Presence of risk related to the use (RRU) of psychoactive substances according to sociodemographic characteristics, living conditions before incarceration and current incarceration of individuals deprived of liberty (n=220). Maringá, PR, Brazil, 2019

Characteristicsof PDLs *	Tobacco products (n=175;79.5%)	Alcoholic Beverages (n=215;97.7%)	Cannabis (n=159;72.3%)	Cocaine and crack (n=132;60.0%)	Amphetamines or ecstasy (n=73;33.2%)	Inhalants (n=93;42.3%)	Hypnotics and sedatives (n=71;32.3%)	Hallucinogens (n=80;36.4%)	Opioids/opiates (n=9;4.1%)
	n (%)	n (%)	n (%)	n (%)	n (%)	n (%)	n (%)	n (%)	n (%)
**Sociodemographics**									
**Age**									
19 to 39 years	86 (49.1)	113 (52.6)	86 (54.1)	72 (54.5)	34 (46.6)	51 (54.8)	48 (67.6)	40 (50.0)	3 (33.3)
40 to 64 years	89 (50.9)	102 (47.4)	73 (45.9)	60 (45.5)	39 (53.4)	42 (45.2)	23 (32.4)	40 (50.0)	6 (66.7)
Skin color									
White	57 (32.6)	79 (36.7)	54 (34.0)	47 (35.6)	29 (39.7)	34 (36.6)	22 (31)	27 (33.8)	6 (66.7)
Other	118 (67.4)	136 (63.3)	105 (66.0)	85 (64.4)	44 (60.3)	59 (63.4)	49 (69)	53 (66.3)	3 (33.3)
**Has a partner**									
No	84 (48.0)	108 (50.2)	78 (49.1)	62 (47)	35 (47.9)	44 (47.3)	42 (59.2)	34 (42.5)	0 (0.0)
Yes	91 (52.0)	107 (49.8)	81 (50.9)	70 (53)	38 (52.1)	49 (52.7)	29 (40.8)	46 (57.5)	9 (100.0)
**Professes a religion**									
No	6 (3.4)	10 (4.7)	7 (4.4)	7 (5.3)	3 (4.1)	4 (4.3)	4 (5.6)	3 (3.8)	2 (22.2)
Yes	169 (96.6)	205 (95.3)	152 (95.6)	125 (94.7)	70 (95.9)	89 (95.7)	67 (94.4)	77 (96.3)	7 (77.8)
Children									
No	59 (33.7)	74 (34.4)	56 (35.2)	44 (33.3)	21 (28.8)	34 (36.6)	30 (42.3)	26 (32.5)	1 (11.1)
yes	116 (66.3)	141 (65.6)	103 (64.8)	88 (66.7)	52 (71.2)	59 (63.4)	41 (57.7)	54 (67.5)	8 (88.9)
**Living conditions before incarceration**									
**Housing condition**									
Owned	99 (56.6)	130 (60.5)	93 (58.5)	77 (58.3)	52 (71.2)	52 (55.9)	41 (57.7)	47 (58.8)	7 (77.8)
Rented	63 (36)	72 (33.5)	55 (34.6)	45 (34.1)	20 (27.4)	32 (34.4)	26 (36.6)	28 (35)	2 (22.2)
Other	13 (7.4)	13 (6)	11 (6.9)	10 (7.6)	1 (1.4)	9 (9.7)	4 (5.6)	5 (6.3)	0 (0.0)
Lived alone									
No	142 (81.1)	178 (82.8)	131 (82.4)	105 (79.5)	58 (79.5)	68 (73.1)	61 (85.9)	64 (80.0)	8 (88.9)
Yes	33 (18.9)	37 (17.2)	28 (17.6)	27 (20.5)	15 (20.5)	25 (26.9)	10 (14.1)	16 (20.0)	1 (11.1)
**Worked**									
No	23 (13.1)	23 (10.7)	23 (14.5)	20 (15.2)	5 (6.8)	16 (17.2)	8 (11.3)	13 (16.3)	0 (0.0)
Yes	152 (86.9)	192 (89.3)	136 (85.5)	112 (84.8)	68 (93.2)	77 (82.8)	63 (88.7)	67 (83.8)	9 (100.0)
Family income									
Without income	13 (7.4)	15 (7)	12 (7.5)	12 (9.1)	6 (8.2)	8 (8.6)	6 (8.5)	7 (8.8)	1 (11.1)
With income	162 (92.6)	200 (93)	147 (92.5)	120 (90.9)	67 (91.8)	85 (91.4)	65 (91.5)	73 (91.3)	8 (88.9)
**Family member responsible until age 15**									
Both parents	54 (30.9)	79 (36.7)	48 (30.2)	44 (33.3)	24 (32.9)	25 (26.9)	19 (26.8)	22 (27.5)	5 (55.6)
Only one of the parents	92 (52.6)	98 (45.6)	83 (52.2)	63 (47.7)	33 (45.2)	47 (50.5)	38 (53.5)	41 (51.3)	3 (33.3)
Others	29 (16.6)	38 (17.7)	28 (17.6)	25 (18.9)	16 (21.9)	21 (22.6)	14 (19.7)	17 (21.3)	1 (11.1)
**Age of first detention**									
12 to 17 years	49 (28)	51 (23.7)	47 (29.6)	45 (34.1)	21 (28.8)	32 (34.4)	20 (28.2)	31 (38.8)	2 (22.2)
18 years or more	126 (72)	164 (76.3)	112 (70.4)	87 (65.9)	52 (71.2)	61 (65.6)	51 (71.8)	49 (61.3)	7 (77.8)
**Current incarceration**									
**Reason for detention**									
Dealing^†^	69 (39.4)	86 (40)	61 (38.4)	45 (34.1)	30 (41.1)	31 (33.3)	26 (36.6)	27 (33.8)	3 (33.3)
Others	106 (60.6)	129 (60)	98 (61.6)	87 (65.9)	43 (58.9)	62 (66.7)	45 (63.4)	53 (66.3)	6 (66.7)
**Prison time**									
Up to one year	124 (70.9)	154 (71.6)	115 (72.3)	96 (72.7)	48 (65.8)	70 (75.3)	49 (69.0)	55 (68.8)	5 (55.6)
More than one year	51 (29.1)	61 (28.4)	44 (27.7)	36 (27.3)	25 (34.2)	23 (24.7)	22 (31.0)	25 (31.3)	4 (44.4)
**Visitation**									
No	73 (41.7)	84 (39.1)	65 (40.9)	57 (43.2)	21 (28.8)	39 (41.9)	29 (40.8)	30 (37.5)	4 (44.4)
Yes	102 (58.3)	131 (60.9)	94 (59.1)	75 (56.8)	52 (71.2)	54 (58.1)	42 (59.2)	50 (62.5)	5 (55.6)

* Persons deprived of liberty; ^†^Dealing in psychoactive substances and/or association to trafficking

The results of the univariate logistic regression models of sociodemographic variables, living conditions before incarceration, and current incarceration on the outcome RRU (present or absent) for tobacco-derived substances, alcoholic beverages, marijuana, cocaine/crack, amphetamines or ecstasy, inhalants, hypnotics/sedatives, hallucinogens, and opioids/opiates are shown in Table 4. 

For the univariate models, there was a significant association of the variables: age (years) with the presence of RRU of hypnotics and sedatives (OR=0.39; CI=0.22;0.71) and opioids/opioids (OR=1.06; CI=1.01;1.12); of skin color with the use of tobacco derivatives (OR=3.1; CI=1.58;6.10) and marijuana (OR=1.88; CI=1.04;3.43); professes religion with tobacco derivatives (OR=4.33; CI=1.33;14.16), alcoholic beverages (OR=13.67, CI=2.04; 91.23) and opioids/opiates (OR=0.17; CI=0.03;0.95); other housing condition with the use of amphetamines or ecstasy (OR=0.12; CI=0.02;0.93); living alone with the use of inhalants (OR=3.22; CI=1.55;6.72); working before arrest with the use of marijuana (OR=0.10; CI=0.01;0.75), cocaine and/or crack (OR=0.27; CI=0.09;0.81), inhalants (OR=0.32; CI=0.13;0.79) and hallucinogens (OR=0.44; CI=0.21;0.90); only one parent as a responsible family member until age 15 with the use of tobacco products (OR=6.81; CI=2.79; 16.66), marijuana (OR=3.67; CI=1.84;7.34), inhalants (OR=2.06; CI=1.16;3.81) and hallucinogens (OR=1.93; CI=1.13;3.27); age of first arrest with the use of tobacco products (OR=0.12; CI=0.03;0.51), marijuana (OR=0.18; CI=0.06;0.49), cocaine and/or crack (OR=0.14; CI=0.06;0.35), inhalants (OR=0.34; CI=0.18;0.64), and hallucinogens (OR=0.26; CI=0.14; 0.51); reason for current arrest with cocaine and/or crack (OR=1.77; CI=1.02;3.07) and whether he receives visitors with amphetamine or ecstasy use (OR=1.96; CI=1.07;3.58).

**Table 4:** Gross odds ratio (OR) for the associations between sociodemographic characteristics, living conditions before incarceration and current incarceration and the presence of risk related to the use (RRU) of psychoactive substances in person deprived of liberty (n=220). Maringá, PR, Brazil, 2019

Characteristicsof PDLs*		Tobacco products (n=175;79.5%)	Alcoholic Beverages (n=215;97.7%)	Cannabis (n=159;72.3%)	Cocaine and crack (n=132;60.0%)	Amphetamines or ecstasy (n=73;33.2%)	Inhalants (n=93;42.3%)	Hypnotics and sedatives (n=71;32.3%)	Hallucinogens (n=80;36.4%)	Opioids/opiates (n=9;4.1%)
Variables and Categories		OR^†^ (CI‡95%)	OR^†^ (CI‡95%)	OR^†^ (CI‡95%)	OR^†^ (CI‡95%)	OR^†^ (CI‡95%)	OR^†^ (CI‡95%)	OR^†^ (CI‡95%)	OR^†^ (CI‡95%)	OR^†^ (CI‡95%)
**Sociodemographics**										
Age (years)	-	1.02 (0.99;1.06)	0.98 (0.90;1.05)	0.98 (0.95;1.01)	0.99 (0.96;1.02)	1.02 (0.99;1.05)	0.99 (0.96;1.02)	0.95 (0.92;0.99)	1.02 (0.99;1.05)	1.06 (1.01;1.12)

Skin color	White	Ref.	Ref.	Ref.	Ref.	Ref.	Ref.	Ref.	Ref.	Ref.
	Other	3.1 (1.58;6.10)	-	1.88 (1.04;3.43)	1.31 (0.75;2.28)	0.91 (0.51;1.61)	1.13 (0.65;1.96)	1.59 (0.87;2.89)	1.35 (0.76;2.39)	0.29 (0.07;1.20)
Has a partner	No	Ref.	Ref.	Ref.	Ref.	Ref.	Ref.	Ref.	Ref.	Ref.
	Yes	1.62 (0.83;3.16)	1.49 (0.24;9.07)	1.22 (0.68;2.21)	1.42 (0.83;2.44)	1.16 (0.66;2.03)	1.24 (0.73;2.12)	0.60 (0.34;1.06)	1.65 (0.95;2.88)	-
Professes a religion	No	Ref.	Ref.	Ref.	Ref.	Ref.	Ref.	Ref.	Ref.	Ref.
	Yes	4.33 (1.33;14.16)	13.67 (2.04;91.23)	1.94 (0.59;6.36)	1.08 (0.33;3.50)	1.52 (0.40;5.80)	1.50 (0.44;5.12)	0.95 (0.28;3.27)	1.76 (0.46;6.71)	0.17 (0.03;0.95)
Children	No	Ref.	Ref.	Ref.	Ref.	Ref.	Ref.	Ref.	Ref.	Ref.
	Yes	1.08 (0.55;2.15)	0.48 (0.05;4.34)	0.83 (0.44;1.57)	1.09 (0.62;1.92)	1.44 (0.78;2.64)	0.83 (0.47;1.45)	0.59 (0.33;1.06)	1.12 (0.62;2.00)	4.32 (0.53;35.21)
**Living conditions before incarceration**										
Housing condition	Owned	Ref.	Ref.	Ref.	Ref.	Ref.	Ref.	Ref.	Ref.	Ref.
	Rented	1.91 (0.90;4.04)	0.55 (0.08;4.01)	1.21 (0.64;2.31)	1.11 (0.62;1.98)	0.57 (0.31;1.06)	1.17 (0.66;2.09)	1.2 (0.66;1.20)	1.1 (0.61;1.98)	-
	Other	4.33 (0.55;34.40)	0.20 (0.02;2.35)	1.54 (0.41;5.82)	1.79 (0.53;5.99)	0.12 (0.02;0.93)	2.77 (0.88;8.72)	0.89 (0.26;3.00)	1.00 (0.32;3.17)	-
Lived alone	No	Ref.	Ref.	Ref.	Ref.	Ref.	Ref.	Ref.	Ref.	Ref.
	Yes	1.86 (0.68;5.07)	0.83 (0.08;7.65)	1.09 (0.49;2.40)	1.8 (0.84;3.85)	1.39 (0.68;2.97)	3.22 (1.55;6.72)	0.71 (0.32;1.55)	1.34 (0.66;2.73)	0.59 (0.07;4.84)
Worked	No	Ref.	Ref.	Ref.	Ref.	Ref.	Ref.	Ref.	Ref.	Ref.
	Yes	0.15 (0.02;1.14)	2.09 (0.22;19.48)	0.10 (0.01;0.75)	0.27 (0.09;0.81)	2.02 (0.72;5.64)	0.32 (0.13;0.79)	0.95 (0.39;2.33)	0.44 (0.21;0.90)	- (-)
Family income	Without income	Ref.	Ref.	Ref.	Ref.	Ref.	Ref.	Ref.	Ref.	Ref.
	With income	0.58 (0.13;2.67)	-	0.63 (0.17;2.33)	0.35 (0.10;1.29)	0.73 (0.25;2.13)	0.62 (0.22;1.77)	0.70 (0.24;2.04)	0.63 (0.22;1.81)	0.57 (0.07;4.87)
**Family member responsible until age 15**	Both parents	Ref.	Ref.	Ref.	Ref.	Ref.	Ref.	Ref.	Ref.	Ref.
	Only one of the parents	6.81 (2.79;16.66)	3.72 (0.38;36.47)	3.67 (1.84;7.34)	1.51 (0.83;2.74)	1.21 (0.64;2.28)	2.06 (1.16;3.81)	2.07 (1.07;3.97)	1.93 (1.13;3.27)	0.48 (0.11;2.08)
	Others	1.5 (0.64;3.52)	1.44 (0.14;14.33)	1.8 (0.79;4.11)	1.54 (0.70;3.38)	1.68 (0.76;3.77)	2.66 (1.21;5.84)	1.86 (0.81;4.26)	2.11 (1.08;4.12)	0.41 (0.05;3.59)
Age of first detention	12 to 17 years	Ref.	Ref.	Ref.	Ref.	Ref.	Ref.	Ref.	Ref.	Ref.
	18 years or more	0.12 (0.03;0.51)	-	0.18 (0.06;0.49)	0.14 (0.06;0.35)	0.63 (0.33;1.21)	0.34 (0.18;0.64)	0.67 (0.35;1.28)	0.26 (0.14;0.51)	1.06 (0.21;5.26)
**Current incarceration**										
Reason for detention	Dealing^§^	Ref.	Ref.	Ref.	Ref.	Ref.	Ref.	Ref.	Ref.	Ref.
	Others	1.02 (0.52;2.00)	0.37 (0.04;3.41)	1.19 (0.66;2.17)	1.77 (1.02;3.07)	0.91 (0.51;1.61)	1.58 (0.91;2.75)	1.20 (0.67;2.15)	1.47 (0.83;2.61)	1.32 (0.32;5.43)
Prison time	Up to one year	Ref.	Ref.	Ref.	Ref.	Ref.	Ref.	Ref.	Ref.	Ref.
	Over one year	1.13 (0.54;2.36)	0.59 (0.10;3.64)	0.85 (0.44;1.61)	0.85 (0.47;1.53)	1.49 (0.81;2.74)	0.71 (0.39;1.30)	1.18 (0.64;2.19)	1.22 (0.67;2.23)	2.06 (0.53;7.94)
Visitation	No	Ref.	Ref.	Ref.	Ref.	Ref.	Ref.	Ref.	Ref.	Ref.
	Yes	0.57 (0.28;1.56)	1.04 (0.17;6.35)	0.76 (0.41;1.41)	0.65 (0.37;1.13)	1.96 (1.07;3.58)	0.81 (0.47;1.41)	0.90 (0.50;1.60)	1.11 (0.63;1.95)	0.79 (0.21;3.05)

* Persons deprived of liberty; ^†^Odds ratio; ^‡^Confidence interval; ^§^Dealing in psychoactive substances and/or association to trafficking.

The final logistic regression models fitted for the three groups of variables (sociodemographic, living conditions prior to incarceration, and current incarceration) on RRU (present or absent) for PASs are shown in Table 5.

For the presence of RRU of tobacco products (Model 1), there was a significant association with the variables skin color brown/black/yellow (OR=2.57; CI=1.18;5.62), those who had only one parent as family responsible until age 15 (OR=6.17; CI=2.36;16.61) and first arrest at age 18 or older (OR=0.12; CI=0.03;0.56). The variables age, professes religion, lived alone, worked before arrest, and family member responsible until age 15 were not associated in the univariate analysis (p>0.05), however, they were analyzed in the multiple regression for having p<0.20.

For alcohol RRU (Model 2), significant association was found with the variable professing religion (OR=19.99; CI=2.50;159.80). As for marijuana (Model 3), significant associations were found between marijuana RRU with working/being employed prior to incarceration (OR=0.12; CI=0.02;0.92), only one parent being the caregiver until age 15, indicating that the chance was almost three times greater relative to those whose both parents were caregivers until age 15 (OR=2.93; CI=1.42;6.03). As for the age of first incarceration, when the age of first arrest occurred after 18 years of age, the chance of RRU was lower relative to those who had their first arrest before their 18^th^ birthday (OR=0.19; CI=0.06;0.56). 

Significant associations were found for cocaine/crack use (Model 4) with PDL living alone prior to incarceration. The chance of RRU was more than twice as high relative to those who lived with others (OR=2.27; CI=1.01;5.06) and the age of first incarceration being in the age range of 18 years or older (OR=0.13; CI= 0.05;0.32). 

For the presence of RRU of amphetamines and ecstasy (Model 5), significant associations were observed between the variables other housing status (OR=0.10; CI=0.01;0.85), living alone (OR=2.27; CI=1.02;5.06), age at first arrest being 18 years or older (OR=0.48; CI=0.24;0.96), and receiving visitors in prison (OR=2.00; CI=1.05;3.80). For the presence of RRU of inhalant PASs (Model 6), statistically significant associations were observed with living alone (OR=3.93; CI=1.82;8.49), working before arrest (OR=0.36; CI=0.14;0.93), and age at first arrest being 18 years or older (OR=0.29; CI=0.14;0.56).

For the presence of RRU of hypnotics and sedatives (Model 7), significant association was found with age (OR=0.96; CI=0.93;0.99) and only one parent responsible until age 15 (OR=1.99; CI=1.02;3.85). As for the presence of hallucinogen RRU (Model 8), a significant association was observed with the age of first arrest in the age group of 18 years or older (OR=0.28; CI=0.14;0.55). Model 9, concerning the multiple analysis of the opioid/opioid RRU, was not adjusted because the observed frequency of PDL with present risk was too low (n=9).

**Table 5:** Adjusted models for the associations between sociodemographic characteristics, living conditions before incarceration and current incarceration and the presence of risk related to the use (RRU) of psychoactive substances in person deprived of liberty (n=220). Maringá, PR, Brazil, 2019

Model 1	Categories	β^‡^	RRU^*^ of tobacco products		
Characteristics of PDLs^†^			OR^§^	CI^||^ (95%)	p
Intercept	-	1.4363	-	-	0.3645
Age (years)	-	0.0359	1.04	1.00;1.08	0.0732
Skin Color	Other	0.9449	2.57	1.18;5.62	0.0177
Professes religion	Yes	1.4796	4.39	0.90;2.14	0.0672
Lived alone	Yes	0.9064	2.48	0.78;7.87	0.1246
Worked	Yes	-2.0293	0.13	0.01;16.22	0.0745
Family member responsible until age 15	Only one of the parents	1.8192	6.17	2.36;1.61	0.0002
	Others	-0.1612	0.85	0.30;2.39	0.7600
Age of first detention	18 years or more	-2.1162	0.12	0.03;0.56	0.0070
RQR^¶^: p=0.1807					
Model 2	Categories	β^‡^	RRU^*^ of alcoholic beverages		
Characteristics of PDLs^†^			OR^§^	CI^||^ (95%)	p
Intercept	-	1.8792	-	-	0.0386
Professes religion	Yes	2.9951	19.99	2.50;159.80	0.0048
Housing condition	Rented	-0.6992	0.50	0.06;3.94	0.5080
	Other	-2.3094	0.10	0.01;1.47	0.0933
RQR^¶^: p=0.3719					
Model 3	Categories	β^‡^	RRU^*^ of cannabis		
Characteristics of PDLs^†^			OR^§^	CI^||^ (95%)	p
Intercept	-	3.6297	-	-	0.0023
Skin Color	Other	0.5269	1.69	0.88;3.24	0.1128
Worked	Yes	-2.1359	0.12	0.02;0.92	0.0417
Family member responsible until age 15	Only one of the parents	1.0760	2.93	1.42;6.03	0.0036
	Others	0.1472	1.16	0.47;2.85	0.7483
Age of first detention	18 years or more	-1.6819	0.19	0.06;0.56	0.0029
RQR^¶^: p=0.5899					
Model 4	Categories	β^‡^	RRU^*^ of cocaine and or crack		
Characteristics of PDLs^†^			OR^§^	CI^||^ (95%)	p
Intercept	-	2.6347	-	-	0.0004
Lived alone	Yes	0.8178	2.27	1.01;5.06	0.0461
Worked	Yes	-1.0805	0.34	0.11;1.09	0.0695
Age of first detention	18 years or more	-2.0677	0.13	0.05;0.32	<0.0001
Model 4	Categories	β^‡^	RRU^*^ of cocaine and or crack		
Characteristics of PDLs^†^			OR^§^	CI^||^ (95%)	p
Reason for detention	Others	0.5430	1.72	0.94;3.15	0.0779
RQR^¶^: p=0.6597					
Model 5	Categories	β^‡^	RRU^*^ of amphetamines or extasy		
Characteristics of PDLs^†^			OR^§^	CI^||^ (95%)	p
Intercept	-	-0.4414	-	-	0.2418
Housing condition	Rented	-0.6015	0.55	0.29;1.05	0.0696
	Other	-2.2895	0.10	0.01;0.85	0.0344
Lived alone	Yes	0.8205	2.27	1.02;5.06	0.0449
Age of first detention	18 years or more	-0.7374	0.48	0.24;0.96	0.0390
Receives visitors	Yes	0.6922	2.00	1.05;3.80	0.0349
RQR^¶^: p=0.1419					
Model 6	Categories	β^‡^	RRU^*^ of inhalants		
Characteristics of PDLs ^†^			OR^§^	CI^||^ (95%)	p
Intercept	-	1.3131	-	-	0.0129
Lived alone	Yes	1.3677	3.93	1.82;8.49	0.0005
Worked	Yes	-1.0230	0.36	0.14;0.93	0.0347
Age of first detention	18 years or more	-1.2547	0.29	0.14;0.56	0.0003
RQR^¶^: p=0.9302					
Model 7	Categories	β^‡^	RRU^*^ of hypnotics and/or sedatives		
Characteristics of PDLs^†^			OR^§^	CI^||^ (95%)	p
Intercept	-	0.0411	-	-	0.9411
Age (years)	-	-0.0404	0.96	0.93;0.99	0.0147
Family member responsible until age 15	Only one of the parents	0.6857	1.99	1.02;3.85	0.0427
	Others	0.5843	1.79	0.77;4.17	0.1744
RQR^¶^: p=0.3880					
Model 8	Categories	β^‡^	RRU^*^ of hallucinogens		
Characteristics of PDLs ^†^			OR^§^	CI^||^ (95%)	p
Intercept	-	0.8322	-	-	0.1087
Has a partner	Yes	0.4264	1.53	0.86;2.74	0.1497
Worked	Yes	-0.7467	0.47	0.19;1.16	0.1035
Age of first detention	18 years or more	-1.2687	0.28	0.14;0.55	0.0002
RQR^¶^: p=0.8354					

* Risk related to use; ^†^Persons deprived of liberty; ^‡^Estimative; ^§^Odds ratio; ^||^Confidence interval; ^¶^Randomized quantile residual.

## Discussion

The main findings of this study were: the sociodemographic profile, the frequency of substance abuse use in life, the RRU levels of PASs and the association of sociodemographic variables with the RRU levels of tobacco, alcohol, marijuana, cocaine/crack, amphetamines, inhalants, hypnotics/sedatives, hallucinogens and opioids.

The profile of the PDLs in this study was mostly young adults, imprisoned for the crime of trafficking in PASs, with less than a year of incarceration, recidivists in the prison system, single, black and brown, with religion, income, children and their own homes. The majority of the PDLs are black and brown, young, with a short period of incarceration, mainly for the crime of trafficking in PASs, single, and with children^4^ ^,^ ^30^. The experience of the reality of the prison system by the children of imprisoned parents can contribute to the increased vulnerability to crime and the consequent perpetuation of incarceration for future generations^30^.

The imprisonment experience is a complex process and can cause a high prevalence of mental disorders, endangering the health of those who are incarcerated, perpetrating self-destructive behaviors^4^ ^,^ ^30^. The consumption of PAS may be related to the very socialization in the prison environment, facilitating the insertion of inmates into dominant social groups in prison. In addition, the use of PAS may act as a defense and escape mechanism for the mental health of detainees with presumable psychopathological worsening, since the addiction is maintained even after the completion of the sentence in the resocialization process^4^ ^,^ ^13^.

Given this scenario, we identified a significant prevalence of substance abuse consumption in the lives of PDLs, mainly alcohol, tobacco, marijuana and cocaine/crack. Marijuana is the most commonly consumed illicit substance, corroborating the national and international literature^14^ ^,^ ^31^, with prevalence rates much higher than those observed among the general Brazilian population (marijuana 7.7%, cocaine 3.1%, crack 0.9%)^32^. Marijuana was the most commonly used illicit drug, followed by cocaine/crack and inhalants, and about a quarter had used hypnotics, hallucinogens, opioids and amphetamines or ecstasy, corroborating international studies that point to marijuana as the most commonly used illicit drug among PDLs^33^.

Regarding RRU of SPAs of abuse, moderate marijuana use was identified in this study, with risk related to low age at first arrest. One study found similar results whose marijuana use was reported by 67.5% of PDLs with onset at the age of ten to 15 years^34^. Cannabis is the most commonly consumed illicit substance and can act as a “gateway” to other, heavier drugs^14^. 

Corroborating the findings of this research, a study conducted in France concluded that substance abuse in the prison environment may be related to the high concentration of arrests for PAS-related crimes, low socioeconomic status, and frequent psychiatric disorders in PDLs^14^. Low- and middle-income countries, such as Brazil, may have a prevalence of PAS abuse and dependence of 25% among PDLs^35^. Research conducted in Ethiopian prisons identified that lack of social support, living in urban areas, psychopathy, and family history of substance use are associated with abuse PAS use in PDLs^15^. 

People in prison PAS use tend to have broader mental and social disorders, including lower educational qualifications, lower employment rates, more housing difficulties, poorer physical health, and more behavioral, psychological, and psychiatric problems, compared to other PDLs^33^ ^,^ ^36^. A similar international study found associations of PAS use with mental health and criminal activity, such as the number of drugs used in life, daily drug use in the six months prior to arrest, and being intoxicated when committing the crime related to the current arrest^33^.

Tobacco has long been considered part of the prison culture, and the smoking situation among PDLs is more serious^37^. In this study, a moderate risk for tobacco use was identified related to age at first arrest, skin color, and family member responsible for care until age 15. A North American study demonstrated that adolescents raised by both parents is a protective factor against the use of tobacco, alcohol, and illicit PASs^11^.

These findings corroborate research that identified increased frequency of tobacco use among prisoners on the grounds of coping with the stress associated with incarceration^38^. The increased consumption of PAS by prisoners in Ecuador was also associated with incarceration^39^, portraying the need to address this issue in the prison environment in order to plan efficient and effective actions with PDLs. 

Regarding alcohol, its consumption in the prison environment showed low risk and was associated with the practice of religiosity. The role that religion and spirituality play in the cessation of criminal behavior and the use of PASs is not yet fully understood, but suggests a relatively high importance in substance use in the prison environment, particularly in relation to alcohol and cocaine^40^. Another Brazilian study also found that inhaled cocaine, at moderate and high levels, had a statistically significant association with the variables not professing religion, risky sexual behavior, age 18 to 34 years, and living with a drug user^41^.

The family context emerges as preponderant in the discussion of substance abuse and the family emerges as the first circle of socialization, internalization of emotions and behaviors that will be experienced in other environments. A study conducted in Greek prisons also found associations between sociodemographic variables with the consumption of PAS as the beginning of consumption at early ages, low education and performance of work activities^34^.

The use of injectable PASs leads the individual to an increased risk of contracting infectious diseases, such as hepatitis C and human immunodeficiency virus. In this study, the use of injectable PASs was lower when compared to other international studies^14^ ^,^ ^42^. However, an increased prevalence of drug use during incarceration was observed. Approximately 15% of PDLs used medications in prison, showing that the main trend was an increase in the consumption of controlled drugs and a decrease in the consumption of other illicit substances of abuse, used as justifications to forget the condition of incarceration^14^ ^,^ ^42^.

A similar study on factors associated with drug use in prisons in Norway showed that, after adjustments on the sociodemographic profile, factors related to mental health and criminal activity showed statistical significance to the number of drugs used in life, daily drug use in the six months prior to arrest, and being intoxicated when committing the crime related to the current arrest^33^. 

The continuous use of PASs by PDLs brings great concerns, since they often do not receive adequate treatment in prison, nor after release, they have a higher risk of returning to addiction, feeding back the cycle to the vulnerability to commit new crimes. Given the high prevalence rates of mental disorders and chemical dependence in prison settings, the United Nations Office on Drugs and Crime (UNODC) and the WHO have issued guidelines on treatment, education, aftercare, rehabilitation and social reintegration measures, as alternatives to conviction or punishment for drug possession offenses. It is emphasized that PDLs with severe mental disorders should not be detained but transferred to appropriate health care facilities^43^. The PDLPS presents, as a proposal, the expansion of the guarantee of social rights, representing a significant advance in health care policies for incarcerated people. However, the fact that there is still abuse of PASs in prison settings still portrays a reality far from ideal. 

The limitation of this study is its cross-sectional design, and it is not possible to establish temporality or causality. Another limitation is due to the fact that specific variables related to mental disorders were not included, addressing only those related to chemical dependence. 

## Conclusion

In this study, PDLs showed high prevalence of PASs use in life, and the risks related to use were moderate for tobacco and marijuana in the prison environment. The results pointed out the importance of developing actions aimed at the problem of PASs use in the prison environment and inserting effective treatment proposals, reducing the gaps and social vulnerability existing in prison.

Health promotion for PDLs is a great challenge for rulers and should be encouraged by public policies. Incarceration may represent an opportunity to identify people who have a history of PASs use from the moment of their admission to the prison unit. The importance of advancing in new studies of marginalized and understudied groups, such as PDLs, is highlighted, thus to strengthen and expand public health policies and understand social inequalities in health.
